# Spatial Ecology of Bacteria at the Microscale in Soil

**DOI:** 10.1371/journal.pone.0087217

**Published:** 2014-01-28

**Authors:** Xavier Raynaud, Naoise Nunan

**Affiliations:** 1 Sorbonne Universités, UPMC Univ Paris 06, Institute of Ecology and Environmental Sciences – Paris, Paris, France; 2 CNRS, Institute of Ecology and Environmental Sciences – Paris, Campus AgroParisTech, Thiverval-Grignon, France; University of Catania, Italy

## Abstract

Despite an exceptional number of bacterial cells and species in soils, bacterial diversity seems to have little effect on soil processes, such as respiration or nitrification, that can be affected by interactions between bacterial cells. The aim of this study is to understand how bacterial cells are distributed in soil to better understand the scaling between cell-to-cell interactions and what can be measured in a few milligrams, or more, of soil. Based on the analysis of 744 images of observed bacterial distributions in soil thin sections taken at different depths, we found that the inter-cell distance was, on average 12.46 µm and that these inter-cell distances were shorter near the soil surface (10.38 µm) than at depth (>18 µm), due to changes in cell densities. These images were also used to develop a spatial statistical model, based on Log Gaussian Cox Processes, to analyse the 2D distribution of cells and construct realistic 3D bacterial distributions. Our analyses suggest that despite the very high number of cells and species in soil, bacteria only interact with a few other individuals. For example, at bacterial densities commonly found in bulk soil (10^8^ cells g^−1^ soil), the number of neighbours a single bacterium has within an interaction distance of ca. 20 µm is relatively limited (120 cells on average). Making conservative assumptions about the distribution of species, we show that such neighbourhoods contain less than 100 species. This value did not change appreciably as a function of the overall diversity in soil, suggesting that the diversity of soil bacterial communities may be species-saturated. All in all, this work provides precise data on bacterial distributions, a novel way to model them at the micrometer scale as well as some new insights on the degree of interactions between individual bacterial cells in soils.

## Introduction

The application of novel molecular techniques (such as high throughput sequencing) during the past two decades has uncovered a phenomenal bacterial diversity in soils. For example, a single gram of soil can harbour up to 10^10^ bacterial cells and an estimated species diversity of between 4·10^3^ [1] to 5·10^4^ species [2]. Several studies have identified major environmental influences on soil bacterial diversity (such as soil pH [3], nitrogen [4], plant communities [5] or land use [6]) and soil bacterial biomass (soil organic carbon [7]), that vary between geographical regions and across biomes. It is intriguing however, that experiments manipulating microbial diversity have found no or only weak links between diversity and many important microbial-driven processes, such as soil carbon mineralization [8]–[10], nitrite oxidation [8], [9] or denitrification [8], [9]. This lack of relationship raises the question about the importance of microbial diversity for soil and ecosystem functioning [11] and has even lead some authors to question the value of studying the soil metagenome for understanding soil microbial functioning [12].

The diversity of biological components can affect ecosystem processes through interactions among species, such as when there is competition for resources, mutualism or predation. The extent and intensity of these interactions depend not only on the interacting species but also on their proximity to one another. The role of space in ecosystem function is widely recognised in higher plant and animal ecology: the spatial distribution of species and the spatial organisation of communities regulate the extent to which individuals interact, such as in competition for resources [13]–[15], mutualism [16] or predation [17], [18], which, in turn, affects ecosystem properties [19]. However, compared to the vast amount of studies focusing on microbial diversity in soils, relatively little attention has been paid to spatial aspects of ecology in microbial systems at the scales at which cell-to-cell interactions occur although there have been some attempts to characterize the spatial distribution of diversity and microbial processes at the scale of aggregates [20]–[22]. As microbial-driven ecosystem processes are sums of the activities of microbial cells, most of which are subject to cell-to-cell interactions, such interactions are likely to have significant effects on overall processes.

In microbial systems, the scale at which individuals interact is related to the distance over which they can effect changes in the concentration of gases or solutes. This may vary depending on the gas or solute and the concentration at which it has an effect on bacterial physiology, however, two notable studies have suggested that the vast majority of interactions occur within 20 µm of bacterial cells [23], [24]. Studies on microbial systems (whether they focus on microbial activity or diversity) are generally carried out at scales many orders of magnitude larger than those at which microorganisms interact with other organisms or with their surrounding environment [25]. This disparity of scale is not as prevalent in the study of higher organisms [26] and so the effects of local interactions on ecosystem processes are better understood. In soil microbial ecology, the effects of local interactions are likely to be obscured by relatively large samples that encompass environmental heterogeneity and local interactions [25].

Microbial-scale processes and local spatial organisation are known to be significant regulators of microbial community stability, function and evolution. Spatial separation has been identified as playing a major role in several microbial processes: 1. it is thought to be responsible for the emergence and maintenance of high levels of bacterial diversity observed in structured media [27]; 2. the relative importance of horizontal gene transfer in bacterial evolution is believed to depend on the proximity of bacterial neighbours, with areas of low cell density dominated by clonal reproduction and densely populated areas harbouring communities in which horizontal gene transfer can be significant [28] and 3. it has been shown that the stability of bacterial communities can depend on the distance among constituent members [29]. A common feature of these bacterial community ecology studies is that they are carried out in model or artificial systems rather than *in situ* and, as a result, the pertinence of the processes identified for real communities in their natural habitats can be questioned. An understanding of the importance of space in bacterial ecology requires knowledge of the distribution of bacterial cells in their environment. This paper aims to explore the spatial distribution of soil bacterial cells in soils at the micrometer scale. To this end, we present a method to analyse and model distributions of individual bacterial cells in soil in order to better understand how bacteria interact with one another. Measuring the distribution of bacterial cells in volumes of soil that are relevant to cell-to-cell interactions is not technically possible at present, therefore our analysis was carried out in three steps. We first studied the distributions of bacterial cells measured in 2 dimensional thin sections of soil and then extended these observations to 3 dimensions using point pattern modelling methods. Finally, we used a simple species abundance model to gain some insight into the degree to which different bacterial species may interact with each other.

## Materials and Methods

### Ethics statement

The Scottish samples on which this study was based were obtained from land belonging to the Scottish Crop Research Institute when one of the authors was a member of staff there and permission was granted. The French samples were given to the authors by Geneviève Grundman of the University of Lyon. However, no new sampling took place for this study. Only previously obtained data were used, most of which has already been published [30]–[32]. The samples were taken from agricultural fields and did not involve any endangered species. Dataset with bacterial distributions is available from the authors upon request.

### Bacterial distribution data

**Figure 1:**
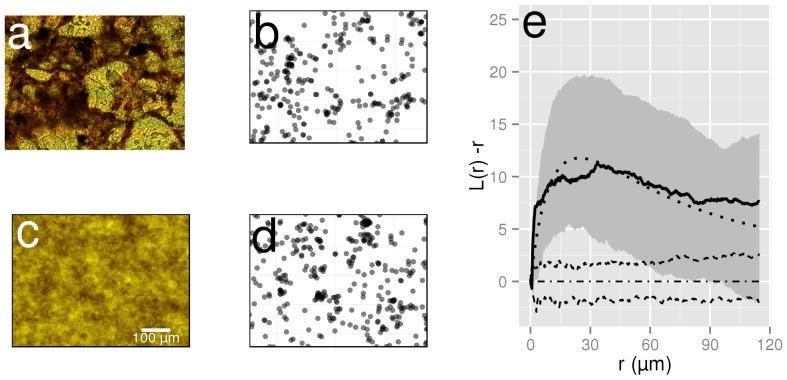
Bacterial habitat, observed and simulated distribution of bacteria in a soil thin section. (a) Bright field image of a soil thin section showing various soil features that characterise the soil microbial habitat and (b) bacterial distribution observed in the same thin section. (c) Random probability field generated using parameters estimated from b (μ = −7.64; σ^2^ = 2.0 and β = 12.95) and (d) simulated bacterial distribution using c. as random intensity (LGCP model). Colours for the random field were chosen to match those of the thin section image. Darker shades indicate higher probability of bacterial presence. The scale for all panels is identical and is indicated in c. (e) Transformed Ripley functions L(r) - r for the observed bacterial distribution shown in a. (solid line) and envelopes of 99 simulations under CSR and LGCP. The dashed lines indicate the envelope of CSR simulations of the same intensity as a. and the shaded area the envelope of simulations of an LGCP with parameters estimated from the observed point distribution. The dotted line corresponds to the theoretical functions under LGCP and the dashed-dotted line to the theoretical function under CSR. In e., L(r)-r above 0 indicates that the point pattern is more aggregated than a random process.

Bacterial distributions in this study consisted of 2D point patterns (the *x* and *y* coordinates of individual cells) measured in images of soil thin sections (Fig. 1). We used 752 new or previously measured bacterial distributions taken from 94 soil thin sections sampled at different depths from a Scottish sandy loam (723 images, [30]–[33]) and 2 soil thin sections of surface soil (20 cm below surface) a French sandy loam (29 images; Table 1). The Scottish samples were taken from topsoil (0–30 cm, sandy loam: 71% sand, 19% silt, 10% clay, pH_H2O_ 6.2, 1.9% C and 0.07% N) and subsoil (30–80 cm, sandy loam: 72% sand, 17% silt, 11% clay, pH_H2O_: 6.5, 0.68% C and 0.02% N) of an arable soil. The French samples were taken from topsoil of an arable soil (0–30, loam: 47.7% sand, 35.3% silt, 17.0% clay, pH_H2O_: 7.0, 1.4% C and 0.13% N). Digital images were acquired with a Zeiss Axioplan 2 microscope fitted for epifluorescence and were equivalent to an effective area of 620×460 µm^2^ for the Scottish samples and 516×410 µm^2^ for the French samples (different cameras were used). Details on the image analysis procedures to extract bacterial coordinates from digital images can be found in [30]. Bacterial distributions that contained 5 cells or less (n = 8) were discarded for the analysis of the spatial distribution of cells, as low cell densities make the analysis of point patterns unreliable. Two thin sections (2×8 microscopic observations) were prepared from a single sample so that one was orthogonal to the other (i.e., XY and XZ). These observations were used to determine whether bacterial distributions at the micro- to millimetre scales were isotropic or not.

**Table 1:** General properties of all observed distribution maps of bacteria at different depths in soil.

Site	Depth (cm)	# samples	Cell density(mm^−2^)	Cell density(g_soil_^−1^)	Average distance to nearest neighbour (µm)
Scotland	0–30 cm	359	668.7±568.0	1.03 10^9^±8.74 10^8^	12.12±6.06
			(17.7–3572.8)	(2.73 10^7^–5.50 10^9^)	(0.38–366.17)
Scotland	30–60 cm	261	297.3±289.5	4.57 10^8^±4.45 10^8^	18.37±11.05
			(17.7–1821.8)	(2.72 10^7^–2.80 10^9^)	(0.45–366.75)
Scotland	>60 cm	103	121.2±165.5	1.86 10^8^±2.54 10^8^	28.94±23.98
			(7.1–1003.1)	(1.09 10^7^–1.54 10^9^)	(0.66–532.36)
France	20 cm	29	3531.28±1809.7	5.43 10^9^±2.78 10^9^	4.95±0.96
			(155.6–7539.1)	(2.39 10^8^–1.16 10^10^)	(0.34–221.12)
	Total	752	576.8±841.0	8.87 10^8^±1.29 10^9^	12.46±9.38
			(7.1–7539.1)	(1.09 10^7^–1.16 10^10^)	(0.34–532.36)

Data for cell numbers, cell densities and average distance to nearest neighbour are given as mean±sd (range). Cell density in g_soil_^−1^ is calculated assuming a microscope depth of field of 2 µm and a soil density of 1.3 g cm^−3^.

### Spatial model fitting of bacterial distribution observed in thin sections

The aim of this first section is to describe the spatial structure of the observed distributions of bacteria. Each distribution was compared to two different spatial null models. The first null model, Complete Spatial Randomness (CSR) or Poisson process, assumes that the position of one point in the point pattern is independent of the position of the others. The second null model, Log Gaussian Cox Process (LGCP) is a model for aggregated point patterns where the aggregation is caused by some environmental heterogeneity [34]. As bacteria live in the soil pore network, the soil structure (Fig 1a), that determines the architecture of the pore network [35], is an important environmental heterogeneity affecting the distribution of bacteria. LGCP are processes defined in *n*-dimensions and are a form of inhomogeneous Poisson process where the intensity is a Gaussian random measure (Fig. 1c). In this study, LGCP with an exponential covariance function were used. These LGCP are determined by three parameters, the mean (μ), variance (σ) and scale (β) of the Gaussian random measure. The three parameters determine the intensity of the point process (the number of points in the point pattern) and the extent of aggregation. The average intensity, λ, of a LGCP is given by:
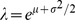
(1)


The CSR model is a special case of LGCP where σ → 0. Details on the theory of LGCP are given in the Appendix S1.

All the point patterns used in this study (observations of bacterial distributions and simulations), were characterized by their intensity λ and 2 summary statistics: Ripley's *K(r)* function and the nearest neighbour distance distribution function, *G(r)* (see [36] for a mathematical definition of these two functions). The intensity λ is the number of points per unit surface and was estimated by dividing the number of points in the distribution by its surface. Ripley's *K(r*) summary statistic is related to the number of points in a point pattern that are within distance *r* of an “average” point. The theoretical expression of Ripley's *K(r)* is known for the CSR and LGCP models. For 2D point patterns, they are given by the following equations:
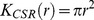
(2)

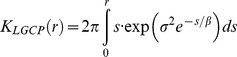
(3)


The theoretical expression of *K(r)* for the LGCP model makes it possible to estimate the parameters μ, σ^2^ and β for all observed bacterial distributions [37]. The nearest neighbour distance distribution function, *G(r)*, of a point pattern is the distribution function of the distance from an average point to its nearest neighbour. The theoretical expression of *G(r)* for a 2D Poisson process is
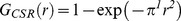
(4)where λ is the intensity of the point process. There is no theoretical expression for *G(r)* for a LGCP process.

Estimates of the two summary statistics and of the LGCP parameters were all carried out using R 2.15 with packages *spatstat* [38] and *Randomfield* [39]. These packages provide all the necessary functions to manipulate and analyse point patterns. We used the Ripley isotropic correction for estimates of *K(r)* and Kaplan-Mayer estimator for *G(r)* [38].

The goodness of fit between each observed distribution (see example in Fig 1b) and the null models (see example in Fig 1d for the LGCP null model) was carried out as in [40]. To begin with, we simulated 99 point processes of the same intensity under the two null models and calculated the estimated *K(r)* and *G(r)* functions for each simulation. For both the simulations and the observed distributions, we then computed the maximum absolute difference between the estimated summary statistics *K(r)* or *G(r)* and its theoretical counterparts to build a two-sided test of significance at *P* = 0.01 which was used to reject the hypothesis that the observed pattern followed the null model. Because there is no theoretical expression of *G(r)* for the LGCP model, the average of the 99 simulations was used as a theoretical counterpart. In the results section, the transformation 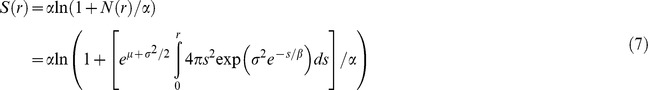
was plotted as it is simpler to interpret (e.g., *L(r)-r* = 0 for all *r* in the case of a 2D CSR process).

### Modelling bacterial neighbourhoods in 3 dimensions

As we found that the LGCP model was adequate for modelling bacterial cell distributions in 2D (see Results section), a similar modelling approach, based on LGCP, was used to estimate the number of neighbours a single bacterial cell had within a given distance in 3 dimensions. The expected values of the parameters μ, σ^2^, β of an isotropic Gaussian random measure of a 3D LGCP are the same as those of a plane from the same Gaussian field [41], [42]. Therefore, we used the 2D estimates of the LGCP parameters to simulate 3D distributions after ascertaining that bacterial distributions were isotropic at the bacterial neighbourhood scale. Simulations of LGCP 3D distributions were carried out following the method given in [34]. Average parameter values corresponding to bacterial densities of ca. 10^8^ cells g^−1^ (μ = −10.26, σ^2^ = 2.90, β = 20), 10^9^ cells g^−1^ (μ = −7.52, σ^2^ = 1.90, β = 25) and 10^10^ cells g^−1^ (μ = −4.91, σ^2^ = 1.29, β = 25) were used to simulate 39 cubes (300×300×300 µm^3^) for each bacterial density. The number of cells each bacterium had in its neighbourhood as a function of distance was calculated, and the average across the 39 simulations determined. This was compared with the theoretical number of neighbours for a 3D LGCP, given by the following equation (see Appendix S1 for details):
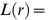
(5)where μ, σ^2^, and β are the parameters of the LGCP. In our simulations the realised densities were (mean±s.e.) 1.06 10^8^±2.48 10^6^, 1.04 10^9^±2.44 10^7^ and 1.02 10^10^±2.47 10^8^ cells g^−1^, respectively.

### Diversity in the bacterial neighbourhood

In order to study the number of bacterial species in the bacterial neighbourhood (i.e., the number of species with which a bacterium might interact), 3D bacterial distributions in which bacterial cells were attributed a species identity were simulated. However, the spatial structure of bacterial diversity at the micrometer scale is unknown, so a modelling approach was taken to estimate the number of species a single bacterium interacts with. As the microbial diversity in our soil samples was not known, we used published data of soil microbial diversity [1], [2] to simulate the 3D bacterial communities. To do so, a simple species-abundance model, the log-series distribution of species [43], was used to calculate the number of individuals per species from a total number of individuals and species. This species abundance model has been found to fit bacterial species distributions in soils at larger spatial scales [44] and has a very simple mathematical formulation. The Fisher species abundance curve relates the number of species (*S*) to the number of individuals (*N*) as described in the following equation:
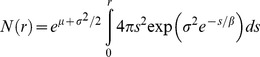
(6)where α is Fisher's α index of diversity. Values for α were calculated using Eq 6 and data found in [1] (S = 4000, N = 1.5 10^10^) or [2] (Agricultural soil, Brazil: S = 3559, N = 10^9^; Forest soil, Canada: S = 15188, N = 10^9^; Agricultural soil, Florida, USA: S = 4477, N = 10^9^; Agricultural soil, Illinois, USA S = 4010, N = 10^9^). This gave values of α of 221.86, 233.04, 1107.53, 297.94 and 264.79, respectively. Because the spatial structure of species at such scales is unknown, species identities were assigned at random among individuals in the simulations. In this case, the average number of neighbouring species can be derived from the theoretical number of neighbours (Eq. 5) and the Fisher species abundance curve (Eq. 6) following the equation:
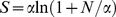
(7)


It should be noted that this type of modelling approach is rather simplistic and was only used to illustrate how interactions between different bacterial species might affect soil functioning (see Discussion).

## Results

### Bacterial densities in thin sections

The number of bacterial cells in the analysed images ranged from 5 to 1599. This corresponded to densities between 7 and 7539 cells mm^−2^ or ca. 1.09 10^7^ to 1.16 10^10^ cells g^−1^ soil, assuming a microscope depth of field of 2 µm and a soil density of 1.3 g cm^−3^ (Table 1). On average, bacterial densities decreased with depth, with the highest densities found above 30 cm depth and the lowest below 60 cm. However, the variability in cell density was high at all depths (Table 1), indicating that bacterial cells were distributed in a heterogeneous way throughout the soil volume. Due to the decrease in density associated with depth, inter-cell distance also varied with depth, with an average of approximately 10 µm at depths of 0–30 cm (corresponding to high cell densities) and 29 µm at depths below 60 cm (Table 1). It should be noted that inter-cell distances were also highly variable at each depth.

### Comparison of observed distributions with the null models

The goodness of fit between the measured distributions and the CSR model revealed clear deviations from CSR (*P*<0.05) in at least 630 of the 744 distributions (Table 2). Clustering was more pronounced in the surface layers of the soil, where bacterial cells were more abundant. Whereas in the surface layers, to a depth of 30 cm, at least 366 of the 387 distributions (94.6%) differed from CSR, only 52 of 98 distributions (53.1%) did so in soil taken from depths below 60 cm (Table 2). An example of the deviation of the Ripley's *K(r)* function of an observed distribution from CSR is given in Fig 1e.

**Table 2:** Total number of samples, number (proportions %) of samples deviating from CSR and number (proportions %) of samples deviating from the LGCP model for different soil depths for all bacterial distribution having more than 5 bacterial cells in the field of view.

Depth	# samples	samples deviating from CSR		samples deviating from LGCP	
		Ripley's K	G function	Ripley's K	G function
0–30 cm	387	376 (97.1%)	366 (94.6%)	37 (9.6%)	97 (25.1%)
30–60 cm	259	228 (88.0%)	212 (81.8%)	14 (5.4%)	40 (15.4%)
>60 cm	98	68 (69.4%)	52 (53.1%)	4 (4.1%)	11 (11.2%)
Total	744	672 (90.3%)	630 (84.68%)	55 (7.4%)	148 (19.9%)

Deviations are calculated based on a goodness of fit test between summary statistics for each observed distribution (Ripley's K or G function) and the corresponding statistics under the null model (CSR of LGCP). Ripley's K is a summary statistics related to the number of points in a point pattern that are within a certain distance to an average point. The nearest neighbour distance distribution G is the distribution function of the distance from an average point to its nearest neighbour.

In contrast, the LGCP null model adequately described the observed distributions in, at worst, 80% of the cases (Table 2). Estimates of the LGCP parameters μ, σ^2^ and β parameters were in the range [−13.49, −5.81], [0.59, 6.85] and [2.59 10^−3^, 161.18], respectively. Complete spatial randomness is the limit of LGCP when σ^2^ tends to 0 and therefore the values of σ^2^ provide a first indication that the bacterial distributions in these soils ranged from highly aggregated (high σ^2^) to near random (low σ^2^) distributions. The mean (μ) decreased (R^2^ = 0.25, P<0.001) and variance (σ^2^) increased (R^2^ = 0.08, P<0.001) linearly with depth between 30 cm and 80 cm below-ground, but no trends were apparent for depths between 0 and 30 cm. There was no relation between β and depth (data not shown). An example of the concordance between the Ripley's function of an observed distribution and simulations of LGCP distribution is given in Fig 1e. The distributions that did not fit the LGCP null model occurred primarily in topsoil and rhizosphere samples where very high bacterial densities were observed, or in subsoil samples in which individual, isolated colonies were detected.

### Isotropy of bacterial distributions

The isotropic nature of the bacterial distributions was tested using the two slides (2×8 images) that were prepared orthogonally. The number of bacterial cells in these slides ranged from 78 to 1008 for one slide and from 31 to 920 for the other. There were no statistical differences in the number of observed cells between the two slides (*P* = 0.27). The estimates of the LGCP parameters for μ, σ^2^ and β were, respectively, in the ranges [−10.19, −6.51], [1.44, 3.99] and [8.94, 61.65] for one slide and [−10.17, −6.37], [1.29, 2.60] and [7.21, 60.09] for the other. Here also, there were no statistical differences between these estimates for the two slides (*P*>0.16 at least), suggesting that the distribution of bacteria at these micrometer to millimetre scales is isotropic.

### Modelling bacterial distributions in 3D

**Figure 2:**
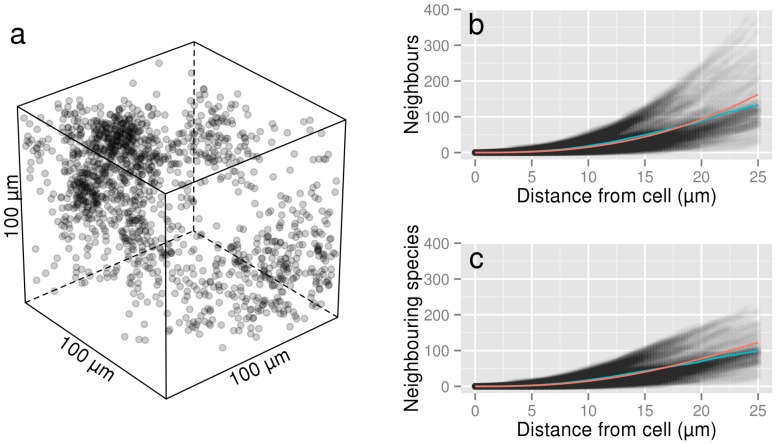
3D simulation of bacterial distribution. (a) 3D representation of bacterial distribution in a 100×100×100 µm^3^ cube as simulated by LGCP. The model parameters for the simulation were μ = −7.52, σ^2^ = 1.90 and β = 25. (b) Number of neighbours as a function of distance for each bacterium (scatterplot), average number of neighbours (red line) and theoretical number of neighbours for the LGCP (blue line) and (c) Number of neighbouring species assuming a random distribution of species among individuals (line colours are the same as in b). The number of species considered in this simulation was S = 450, corresponding to Fisher's α = 221.86 (estimated from [1]).

**Figure 3:**
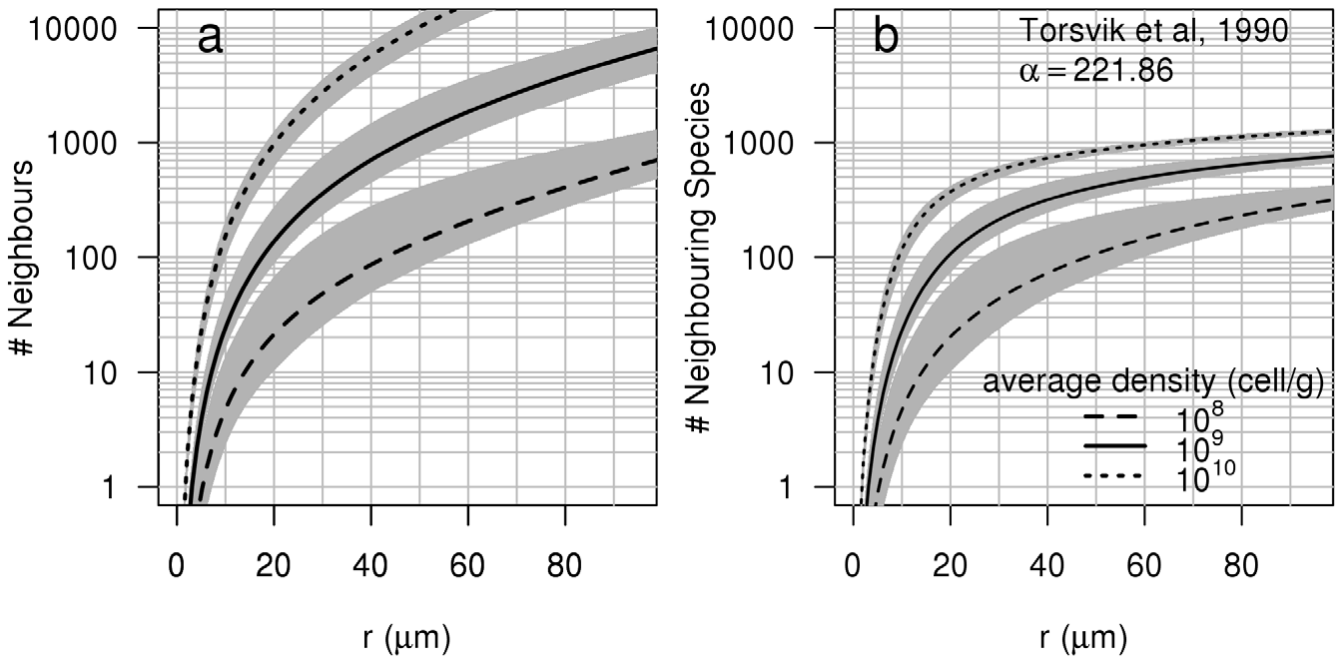
Number of neighbours and neighbouring species. (a) Mean number of neighbours and (b) neighbouring species around an “average” bacterium as a function of distance from the bacterium and as a function of bacterial density. The mean number of neighbours and neighbouring species are derived from Ripley's K(r) function of LGCP with parameters μ = −10.26, σ^2^ = 2.90, β = 20 for the 10^8^ cells g^−1^ soil density, μ = −7.52, σ^2^ = 1.90, β = 25 for the 10^9^ cells g^−1^ soil density and μ = −4.91, σ^2^ = 1.29, β = 25 for the 10^10^ cells g^−1^ soil density. Grey envelopes surrounding curves represent the maximum and minimum of these numbers calculated from 39 simulations. Note the logarithmic scale on the y axis.

Due to the apparent isotropic nature of the bacterial neighbourhoods at micrometer to millimetre scales, the LGCP parameters obtained in 2D space were used to simulate 39 independent 3D distributions [41]. An example of such a 3D distribution of bacteria (density equivalent to 10^9^ cells g^−1^) is given in Fig. 2a. For each simulation, the number of neighbours each bacterium had as a function of distance was computed (shaded scatterplot in Fig. 2b) and averaged across all bacteria (red line in Fig. 2b). As expected, the average number of neighbours bacteria had as a function of distance was similar to the theoretical number of neighbours derived from the theoretical expression of *K(r)* for a LGCP (Eq 2, blue line in Fig 2b). The number of neighbours that a single bacterium had within a distance of 20 µm ranged from 7 to 250 for a bacterial density of 10^9^ cells g^−1^ (Fig. 2b). The minimum and maximum average number of neighbours obtained across the 39 simulations are given in Fig 3a. Overall, these results suggest that the number of cells in the neighbourhood of a typical bacterium is rather limited. For an average density of 10^9^ cells g^−1^ soil, the average number of neighbours around a single cell was ca. 1043 (±250) cells within a distance of 50 µm, decreasing to ca. 120 (±40) cells at 20 µm (Fig 3a). Similarly, for an average density of 10^8^ cells g^−1^ soil, the average number of neighbours was ca. 82 (±22) cells within a distance of 50 µm and ca. 12 (±4) cells at 20 µm. Finally, for bacterial densities close to what one would expect in the rhizosphere, (10^10^ cells g^−1^ soil), the average number of neighbours was ca. 5806 (±1000) cells within a distance of 50 µm decreasing to ca. 555 (±100) cells within 20 µm.

### Bacterial diversity in the bacterial neighbourhood

In order to estimate the diversity of bacterial species a typical bacterium interacts with, species were distributed at random among individual cells in the 3D simulations of bacterial distributions. Using these simulations, we estimated the number of species in the neighbourhood of each cell as a function of distance (shaded scatterplot in Fig. 2c) and computed its average value (red line Fig. 2c). The average number of species (*S(r)*) in the neighbourhood of a single cell was adequately approximated by the theoretical average number of species around a single bacterium given in Eq. 7 (red and blue lines in Fig 2c).

The average number of species in the neighbourhood of bacteria was estimated from the 39 3D simulations at densities of 10^8^, 10^9^ and 10^10^ cells g^−1^ soil (Fig 3b). At lower bacterial densities, typical of bulk soil (10^8^ cells g^−1^), the average number of species that a bacterium can be expected to interact with (species within 20 µm, assuming a random distribution of species) was ca. 11 (±4) species. For a density of 10^9^ cells g^−1^ soil, the average number of neighbouring species around a single cell increased to 97 (±24) species. Finally, when bacterial densities were set at values expected in the rhizosphere (10^10^ cells g^−1^ soil), the average number of species within an interaction distance of 20 µm was ca. 284 (±30) species. These values were higher when the interaction distance was set at 50 µm (Fig 3b). The diversity indices derived from Roesch et al (2007) [2] were similar to that derived from Torsvik et al (1990) [1] with one exception: bacterial diversity in the forest soil in Roesch et al (2007) [2] was much higher (α = 1107.53), resulting in a greater number of species per volume of soil (Fig S1). However, despite the increase in the overall number of species, no increase in the number of neighbouring species within 20 µm was observed for bacterial densities of 10^9^ cells g^−1^ soil and lower.

## Discussion

### The distribution of bacteria in soils

Unsurprisingly, the bacterial distributions measured in the 744 soil thin sections studied showed a high degree of aggregation at all depths, although aggregation was more frequent in the surface strata than in the subsoil. This corroborates similar observations by [30] on a sample of this dataset. We also found that bacterial distributions were isotropic at the millimetre scale. Although this was based on a limited number of observed distributions and would need to be confirmed on a larger dataset, such an observation is not surprising as the vertical gradients observed in soils generally occur at larger scales [45], [46].

Log Gaussian Cox Processes (LGCP) were used to characterise the bacterial distributions and construct 3D distributions from these observations. LGCP are particularly useful for modelling aggregated spatial point patterns where aggregation is due to a stochastic environmental heterogeneity [34]; that is to say, an environmental factor that is continuously and heterogeneously distributed in space. They do not, however, account for the contribution of intrinsic processes, such as birth and death, to the distribution of points in a given point pattern. The distribution of bacteria in soil is clearly a consequence of both extrinsic (environmental conditions, such as pore size and organic matter availability) and intrinsic (reproduction by binary fission) processes. However, it is likely that intrinsic processes such as reproduction are related to the extrinsic processes, as the probability of growth is greater where external environmental conditions are most suitable (i.e. presence of organic substrate, O_2_, water…). It is worth noting that the majority of samples that were inadequately described by LGCP were from the rhizosphere or topsoil. There, intrinsic processes (e.g. cell division) would have played a significant role in the generation of these distributions as bacterial growth may be somewhat decoupled from environmental heterogeneity due to the overall high availability of resources. This may possibly be because environmental heterogeneity (with respect to bacterial growth) was reduced due to the input of organic substrate from plants. It can be concluded therefore, that the relative importance of the underlying processes contributing to the generation of the bacterial distributions (intrinsic vs. extrinsic) can change with situation.

Despite the deviation of some of the bacterial distributions from the LGCP model in some specific cases, LGCP were used to simulate bacterial distributions because they have the distinct advantage of being analytically tractable (i.e. the theoretical expression of *K(r)* is known), making it possible to estimate model parameters from which an observed point pattern could have emerged [37].

### The neighbourhood of bacteria in soils

Three properties of microbial communities emerge from our analysis. The first property is that the number of cells within interacting distances in the neighbourhood of a bacterium is, on average, rather limited compared to the number of cells commonly found in a single gram of soil. For densities equivalent to 10^9^ cells g^−1^, the number of neighbours a typical bacterium has within a distance of 20 µm is approximately 120, increasing to 1000 within a distance of 50 µm. The actual number of neighbours within interaction distances may in fact be lower as the calculations carried out here used the euclidian distance between cells rather than the geodesic distance and therefore did not account for pore geometry. For example, two cells on either side of a sand grain will not interact through the sand grain but rather around it.

The second property is that, given the high variability in the number of neighbours around bacterial cells in our dataset (Table 1) and in our simulations (Fig 2 and 3), the density of interactions is highly variable in space, even at very fine scales, with some cells interacting with few others and other cells interacting with many more. For example, some filamentous bacteria or bacteria in colonies or biofilms are likely to have completely different interaction environments to those of isolated individual bacterial cells. Such localized “pockets of interactions” might have important consequences for ecosystem processes and microbial community evolutionary dynamics [47].

The third property to emerge from this analysis, an obvious corollary of low levels of cell-to-cell interactions, is that the number of different species an individual cell interacts with is also limited. The simulations of bacterial distributions in 3D suggest that interspecific interactions in soils are orders of magnitude lower than what is possible in view of the species diversity often measured in soil (4000–50000 species g^−1^ soil; [1], [2]). For densities equivalent to 10^9^ cells g^−1^ soil, we found that the number of bacterial species within interaction distances (<20 µm) ranged from 1 to 120 (±40) and, even at the highest densities (equivalent to 10^10^ cells g^−1^ soil), never exceeded 1000 species (Fig 3b, Fig S1). Moreover, these data are most likely an overestimate of the actual local diversity, as we assumed that species were distributed randomly among individuals. However, as bacteria reproduce by binary fission, this assumption is almost certainly wrong. The approach taken here thus provides only an upper limit to the number of species in the bacterial neighbourhood. Furthermore, the model also assumes that there is a positive relationship between abundance and diversity as the diversity parameter (α) is constant. This assumption may also be untrue. In reality, species are aggregated and the extent of species aggregation is most probably positively related to growth intensity because bacteria grow by binary fission. This means that diversity may not increase monotonically with abundance when conditions are favourable for growth and it has indeed been found that bacterial diversity in the rhizosphere is lower than in bulk soil [48], [49]. A more realistic way to model the spatial distribution of diversity should therefore account for both the environmental determinism of the spatial distribution of cells (as was done here) and cellular reproduction processes in order to yield aggregation in species distribution.

It is noteworthy that the diversity at the microscale (species within 20 µm of a bacterial cell) did not change appreciably as a function of the global diversity (species found in a gram of soil) for bacterial densities of 10^9^ cells g^−1^ soil or less (i.e., diversity at 20 µm is similar in Fig 3b and Fig S1, Forest Soil, despite very different α values), suggesting that local communities may be species saturated [50]. It is now widely recognised that local diversity is not solely dependant on local interactions, but that regional processes are also important (in the case of soil bacteria, a gram of soil can be considered a region [26]). Species saturation of local communities can arise from species interactions (community membership is limited by competitive exclusion or local environmental conditions [50]) or from the physical limitations of the environment (if the local environment can only accommodate 100 individuals then there cannot be more than 100 species regardless of the overall diversity [51]). It has been suggested that the lack of relationship between ecosystem processes and diversity in soils is due to functional redundancy within soil microbial communities. Regardless of the underlying cause for the apparent local species saturation, species saturation of local microbial communities may also explain the relative insensitivity of many ecosystem processes (e.g. soil organic matter decomposition, denitrification), as well as the resistance or resilience of these processes to environmental stresses, to experimental changes in microbial diversity [52], [53]. If the number of species that the average bacterium interacts with does not change as the overall diversity is changed then the functioning the bacteria are responsible for may not be affected by changes in overall diversity either, as the actual levels of diversity in communities remains the same.

Should this hypothesis be confirmed, there are a number of important consequences for soil microbial ecology. The first is that the relationship between diversity and functioning may only be understood if studied at an appropriate scale. The second is that the relationship between regional (gram of soil) and local diversity (microbial neighbourhood) must be understood if the effects of diversity on soil functioning is to be apprehended.

## Conclusions

Our analysis of 744 observations of *in situ* bacterial distributions in soils indicates that bacterial cells are aggregated at the scale of a few micrometers, most likely due to soil structure and the way bacterial cells reproduce. The analysis also suggests that, because cells interact only at very small distances, the number of cells that a typical bacterial cell interact with is relatively limited, as is the number of bacterial species. Such low levels of bacterial interactions could be a reason why several soil microbial processes appear not to be affected during microbial diversity erosion experiments.

## Supporting Information

Figure S1 Number of neighbouring bacterial species around a typical bacterium as a function of distance at different bacterial densities for the four species diversity levels estimated by Roesch et al (2007). Grey envelopes surrounding curves represent the maximum and minimum of these numbers calculated from 39 simulations with the same parameters values as in Fig. 3. Note the similarity in the number of neighbouring species, for all diversity levels, when bacterial density is 10^9^ cells g^−1^ or less. α = 1107.53 corresponds to a species richness of 15000 species for 10^9^ cells whereas α = 264.79 corresponds to a species richness of 4010 species for the same number of cells.
(TIF)

Appendix S1 Notes on the theory of Log Gaussian Cox Processes.
(DOC)
