# Supplementary figures and images for: Spatial Ecology of Bacteria at the Microscale in Soil
Source: PLoS One. 2014 Jan 28;9(1):e87217. doi: 10.1371/journal.pone.0087217 (PMC3905020; doi:10.1371/journal.pone.0087217)

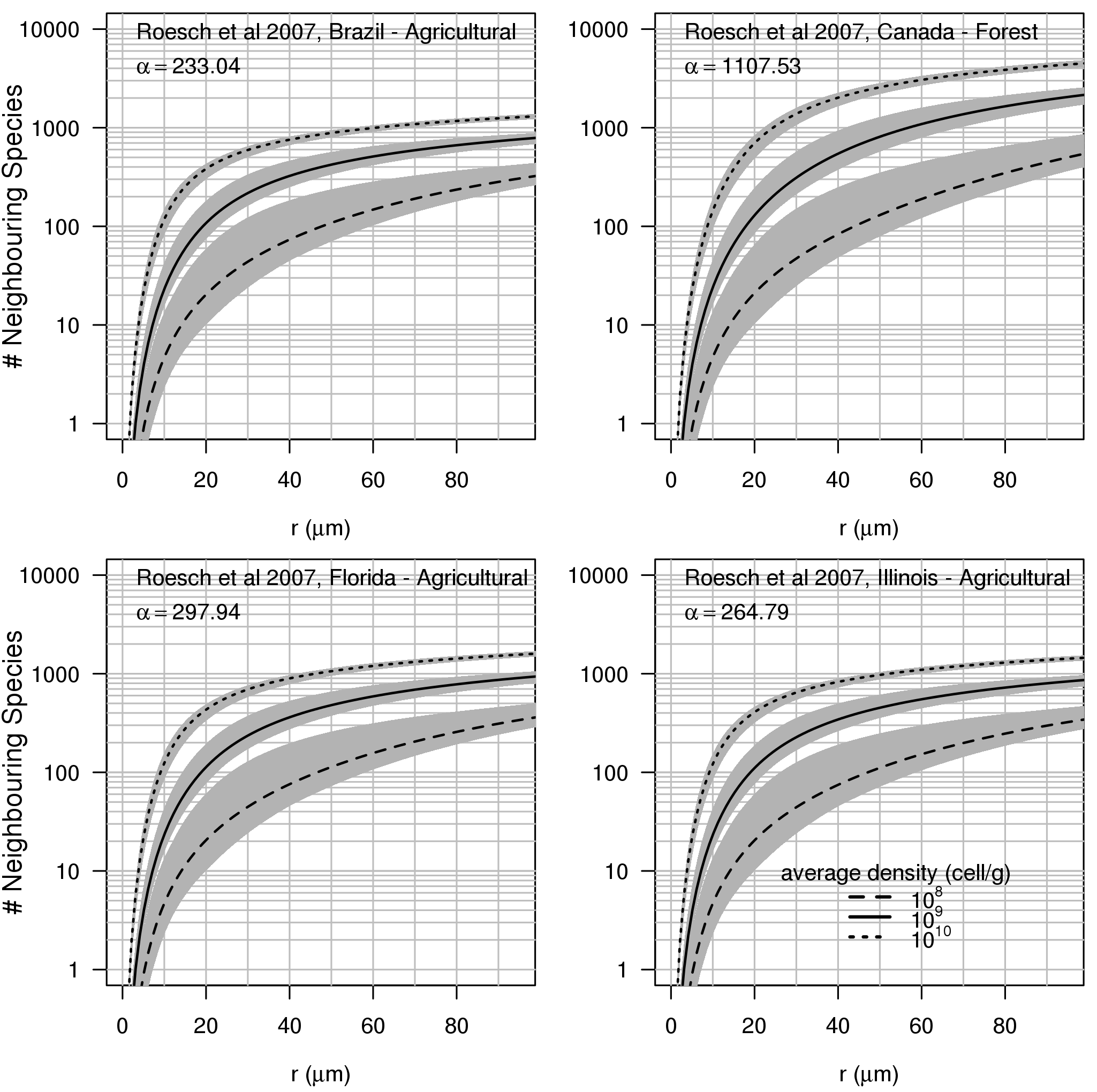

Supplement: Figure S1 — Number of neighbouring bacterial species around a typical bacterium as a function of distance at different bacterial densities for the four species diversity levels estimated by Roesch et al (2007). Grey envelopes surrounding curves represent the maximum and minimum of these numbers calculated from 39 simulations with the same parameters values as in Fig. 3. Note the similarity in the number of neighbouring species, for all diversity levels, when bacterial density is 109 cells g−1 or less. α = 1107.53 corresponds to a species richness of 15000 species for 109 cells whereas α = 264.79 corresponds to a species richness of 4010 species for the same number of cells. (TIF) [file pone.0087217.s001.tif]
