# Supplementary material for: Spatial Ecology of Bacteria at the Microscale in Soil
Source: PLoS One. 2014 Jan 28;9(1):e87217. doi: 10.1371/journal.pone.0087217 (PMC3905020; doi:10.1371/journal.pone.0087217)
Supplement: Appendix S1 — Notes on the theory of Log Gaussian Cox Processes. (DOC) [file pone.0087217.s002.doc]

Notes on the theory of Log Gaussian Cox Processes

Cox processes are models for aggregated point processes where the aggregation is caused by an environmental heterogeneity [1]⁠. A Cox process is said “doubly stochastic” as it arises as an inhomogeneous Poisson process with a random intensity measure. In a Log Gaussian Cox Processes (LGCP) the environmental heterogeneity is modeled by a Gaussian process so that the properties of the LGCP and the Gaussian process are identical. For our study, we have considered Gaussian processes with an exponential covariance function:

$C\left(r\right)=\sigma^{2}e^{-r/\beta}$ (S1)

so that, the Gaussian process (and the LGCP) is solely determined by three parameters, its mean (μ), variance (σ) and scale (β). A homogeneous Poisson process (Complete Spatial Randomness) may be considered as the limit of a LGCP as σ tends to zero.

The intensity λ of a LGCP (the number of points per unit area or volume) is

$\lambda=e^{\mu+\sigma^{2}/2}$ (S2)

LGCP are analytically tractable: the Ripley's K function for a 2D point process is

$K\left(r\right)=\int_{0}^{r} 2\pi se^{C\left(s\right)}ds$ (S3)

whereas, for a 3D point process, it is :

$K\left(r\right)=\int_{0}^{r} 4\pi s^{2}e^{C\left(s\right)}ds$ (S4)

where *C(s)* is the covariance function (Eq. S1).

Combining Eqs S2, S3 and S4 gives the theoretical number of cells around an average cell in 3D

$N\left(r\right)=\lambda*K\left(r\right)=e^{\mu+\sigma^{2}/2}\int_{0}^{r} 4\pi s^{2}\text{exp}\left(\sigma^{2}e^{-r/\beta}\right)ds$ (S5)

1. Møller J, Waagepetersen RP, Syversveen AR (1998) Log Gaussian Cox processes. Scand J Stat 25: 451–482.
